# The prefrontal cortex of homozygous ε3 APOE carriers with local African ancestry has fewer transcripts compared to individuals with local European ancestry

**DOI:** 10.1002/alz.093090

**Published:** 2025-01-03

**Authors:** Nascimento Santos, Michel Satya Naslavsky, Luciana Amaral Haddad, Claudia Kimie Suemoto, Renata Eloah de Lucena Ferretti‐Rebustini

**Affiliations:** ^1^ University of São Paulo, São Paulo Brazil; ^2^ Division of Geriatrics, Department of Internal Medicine, University of Sao Paulo Medical School, São Paulo, São Paulo Brazil; ^3^ Biobank for Aging Studies of the University of São Paulo, São Paulo Brazil; ^4^ University of São Paulo Medical School, São Paulo, São Paulo Brazil; ^5^ Division of Geriatrics, University of São Paulo Medical School, São Paulo, São Paulo Brazil; ^6^ University of São Paulo Medical School, São Paulo Brazil; ^7^ University of Sao Paulo Medical School, São Paulo Brazil; ^8^ Brazilian Brain Bank of the Aging Brain Study Group; University of São Paulo, São Paulo Brazil; ^9^ University of Sao Paulo Medical School, Sao Paulo Brazil; ^10^ Biobank for aging studies of the University of São Paulo, São Paulo Brazil; ^11^ Escola de Enfermagem, Universidade de São Paulo, São Paulo Brazil

## Abstract

**Background:**

he APOE gene has been identified as a major risk factor for late‐onset Alzheimer’s disease (AD) and has three most common alleles: ε2, ε3, and ε4. The presence of the ε4 allele confers a dose‐dependent increased risk for the disease (odds ratio 12.9 for homozygous individuals and between 3.2 and 4.2 for heterozygous individuals). The most prevalent form of Alzheimer’s disease has a multifactorial etiology where factors such as age and sex and the ancestry around the APOE gene is relevant to the disease’s risk. Individuals of African‐American local ancestry have a reduced risk of developing AD compared to European whites and Asians.

**Method:**

this project aims to quantitatively characterize the role of different local ancestry in the different APOE genotypes using 49 post‐mortem frontal cortex tissues with different genotypes and local ancestry combinations (ε3ε3 European‐European, African‐African and ε3ε4 European‐European, African‐African, European‐African, African‐European) and to associate them with the expression of this gene using allele‐specific and non allele‐specific molecular markers and RT‐qPCR techniques.

**Result:**

Within the heterozygous groups, we observed a constant reduced mean expression of the ε4 allele compared with ε3, but these differences did not achieve statistical significance, as didn’t the total APOE expression between these groups with different ancestries. Although we identified a significant difference (p‐value = 0.039) between the homozygous individuals of different ancestry (ε3 European ε3 European versus ε3 African ε3 African), the total expression of individuals of European local ancestry was twice superior to that of African ancestry (European mean: 0.416; African mean: 0.218).

**Conclusion:**

These data support the hypothesis that the lower expression of APOEε3 may be part of the explanation for the reduced African risk of DA in homozygous individuals.

